# Le chondrosarcome naso-sinusien: à propos de deux cas et revue de la literature

**DOI:** 10.11604/pamj.2014.19.165.4643

**Published:** 2014-10-17

**Authors:** Mohamed Mliha Touati, Mehdi Chihani, Youssef Darouassi, Mohammed Lakouichmi, Khalid Tourabi, Brahim Bouaity, Haddou Ammar

**Affiliations:** 1 Service d'Oto-rhino-laryngologie et Chirurgie Cervico-faciale, Hôpital Militaire Avicenne, Marrakech, Maroc; 2 Service de Chirurgie Maxillo-faciale et Plastique, Hôpital Militaire Avicenne, Marrakech, Maroc

**Keywords:** Chondrosarcome naso-sinusien, tumeur maligne, sinus maxillaire, naso-sinusal chondrosarcoma, malignant tumor, maxillary sinus

## Abstract

Le chondrosarcome est une tumeur maligne très destructrice d'origine cartilagineuse, osseuse et mesnchymateuse. La localisation au niveau de la tête et cou est rare et le siège naso sinusien est encore plus rare. Nous rapportons deux observations de chondrosarcome du sinus maxillaire droit et sphéno ethmoïdale. Le but de notre travail est de montrer à travers ces deux cas cliniques, l'intérêt de la tomodensitométrie et de la résonance magnétique dans la présemption diagnostique en corrélation avec la clinique et l'endoscopie,de discuter le choix de la voix et la technique d'abord chirurgical et la surveillance post opératoire. A travers ces deux observations nous soulignerons les difficultés que pose cette tumeur à l'anatomopathologiste pour différencier entre chondrome et chondrosarcome.

## Introduction

Le chondrosarcome est une tumeur maligne très destructrice d'origine cartilagineuse, osseuse et mesnchymateuse [1, 2]. La localisation au niveau de la tête et cou est rare et le siège naso sinusien est encore très rare. Nous rapportons deux observations de chondrosarcome du sinus maxillaire droit et sphéno ethmoïdale.

## Patient et observation

### Observation N°1

**Figure 1:**
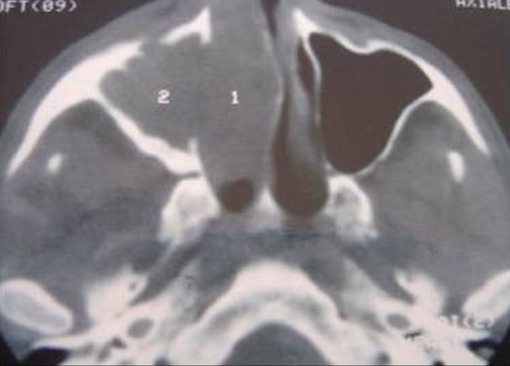
TDM nasosinusienne en Coupe axiale montrant un processus tumoral tissulaire occupant le sinus maxillaire droit lysant la paroi antérolatérale du sinus et repoussant la cloison nasale à gauche

**Figure 2:**
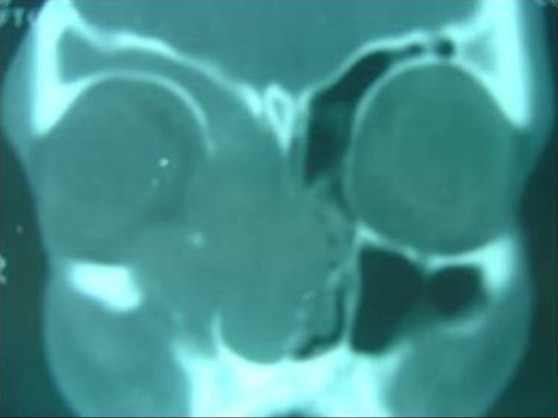
TDM nasosinusienne en coupe coronale montrant, un processus tumoral polylobé, occupant le sinus maxillaire droit et la fosse nasale homolatérale avec extension dans l'orbite droit après lyse de sa paroi médiale et dans la fosse nasale controlatérale après lyse de la cloison nasale

Patient âgé de 65 ans, sans aucun antécédent pathologique notable et qui présentait 6mois avant sa consultation au service ORL, une obstruction nasale unilatérale droite, avec épistaxis de moyenne abondance spontanément résolutives, qui n'a pas inquiété le patient jusqu′à apparition d'un oedème jugale droit, l'examen clinique à l'endoscopie endonasale montre un processus tumoral obstruant la narine droite et arrivant au vestibule, ne saignant pas au contact et couverte de secrétions purulente. L'examen ophtalmologique n'a pas noté d'enophtalmie ou d'exophtalmie. L'examen bucco dentaire et des paires crâniennes est normal. Les aires ganglionnaires sont toutes libres. Un scanner (TDM) crânio facial est fait et montre (Figure 1, Figure 2) un processus tissulaire isodense occupant le sinus maxillaire droit avec lyse osseuse de la paroi antérolatérale du sinus et la lame papyracé, repoussant la cloison nasale a gauche. Nous n'avons pas noté de calcifications évidentes. Une biopsie sous anesthésie locale a été décidée et dont l’étude histhologique a conclue a un sarcome et l’étude immunohistochimie a confirmé le diagnostic de chondrosarcome. Après consultation d'anesthésie et consentement du patient la voie d'abord adoptée est la para-latéro-nasale droite, l'exérèse est totale et la pièce est adréssée à l'anatomopathologiste pour confirmation du diagnostic, les suites sont simples. Le contrôle scânnographique est satisfaisant. Notre patient est ensuite adressé à l'institut national d'oncologie (INO) pour radiothérapie. Notre patient est perdu de vue 6 mois après l'arrêt du traitement.

### Observation N°2

C'est le cas d'une patiente de 57 ans, sans aucun antécédent pathologique particulier, et qui présentait une année avant son admission au service ORL de l'hôpital Militaire Avicenne de Marrakech, une obstruction nasale bilatérale avec rhinorrhée purulente traînante et une épistaxis de petite abondance, associées à une céphalée chronique, la patiente a été traitée à plusieurs reprises comme sinusite maxillaire droite sans nette amélioration. Devant l'apparition d'une tuméfaction de l'angle interne de l’œil droit, la patiente est adressée au service ORL pour prise en charge, l'examen clinique montre une tuméfaction de l'angle interne de l’œil droit sans diplopie ni exophtalmie évidente. L'examen endoscopique endonasal, montrent un processus tumoral de la narine droite issue du méat moyen droit d'aspect blanc rosé non saignant détruisant la cloison nasale et étendu a la narine gauche.

**Figure 3:**
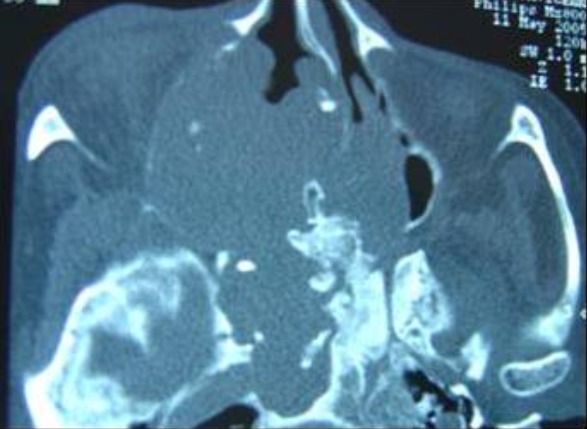
TDM nasosinusienne coupe axiale montrant un processus tissulaire occupant le complexe éthmoïdo sphénoïdal droit étendu au sinus maxillaire homolatérale et à la fosse nasale controlatérale avec lyses osseuses multiples

**Figure 4:**
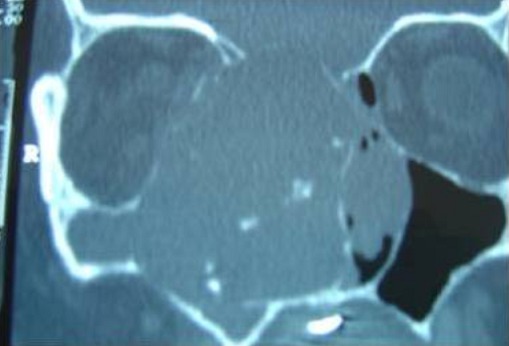
TDM nasosinusienne en coupe coronale montrant le processus tumorale étendu dans l'orbite droit après lyse de sa paroi osseuse médiale et la lyse de la lame criblée de l'ethmoïde. Présence de calcification au niveau de la masse tumorale

**Figure 5:**
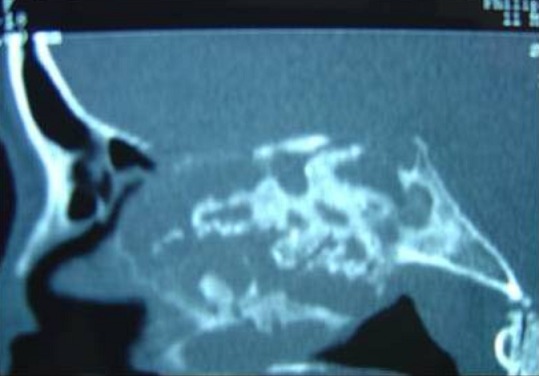
TDM nasosinusienne en coupe axiale, montrant la lyse osseuse étendue du complexe ethmoïdo sphénoïdal et la lyse de la lame criblée de l'ethmoïde sans nette extension endocrânienne

**Figure 6:**
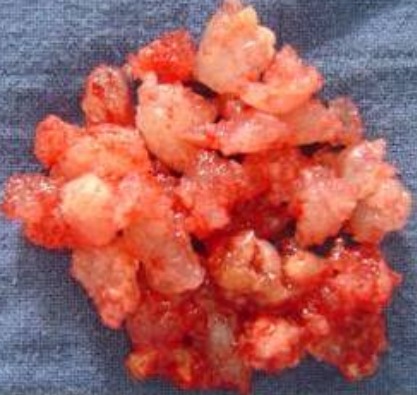
Pièce opératoire dont l'aspect rappelle un tissu cartilagineux

L'examen ophtalmologique ne montre pas de diplopie ni baisse de l'acuité visuelle, l'examen neurologique et notamment des paires crâniennes est normal. Les aires ganglionnaires cervicales sont toutes libres. La TDM est faite et a montrée un processus tumoral de densité hétérogène, occupant le sinus sphénoïdal, l'ethmoïde, et le sinus maxillaire droit avec extension à l'orbite homolatéral et à la narine gauche. Elle a montré aussi une lyse osseuse diffuses et des calcifications au niveau la masse tumorale (Figure 3, Figure 4, Figure 5). Une biopsie a été faite sous anesthésie locale et l’étude histhologique et histhochimique a conclue à un chondrosarcome. Après consultation d'anesthésie et consentement éclairé de la patiente une voie para-latéro-nasale a été décidée, la résection est incomplète vue l'extension a l'orbite et à la base du crâne, la pièce d'exérèse (Figure 6) est adressée a l'anatomopathologiste qui confirme le diagnostic. Les suites sont simples. La patiente a bénéficié d'une chimiothérapie et une radiothérapie, le contrôle clinique et scânnographique a montré un tissu résiduel au niveau orbitaire et sous la lame criblée de l'ethmoïde, la patiente est toujours suivie en consultation ORL, le dernier contrôle remonte au mois de mars 2014.

## Discussion

Le chondrosarcome est un groupe hétérogène des tumeurs malignes d'origine cartilagineuse mais aussi osseuse et mésenchymateuse [1, 2]. La localisation au niveau de la tête et cou est rare, il représente 0,1% des carcinomes de cette région [3], le chondrosarcome des sinus de la face est encore très rare. Les sites habituels du chondrosarcome au niveau maxillo-facial sont les cavités nasales, les sinus para nasaux et la mandibule. La prédominance masculine [4] est notée par plusieurs auteurs, souvent entre la quatrième est la septième décade [5], nos deux patients entre dans cette fourchette d’âge. Les chondrosarcomes originaires du tissu cartilagineux et des tissus mous, sont fréquents chez les hommes après 50 ans et les chondrosarcomes d'origine osseuse sont fréquents chez la femme et les patients moins de 50 ans [3]. Les métastases ganglionnaires et à distance sont rares et représentent respectivement 5,6 et 6,7% [6].

L'expression clinique des chondrosarcomes des sinus de la face est celle de toutes les tumeurs malignes naso sinusiennes, ainsi on peut noter une algie faciale d'intensité variable, des céphalées, une obstruction nasale uni ou bilatérale ou des épistaxis. Selon l'intensité de l'extension aux structures voisines on peut avoir des signes visuels, des anomalies dentaires ou des signes neurologiques par atteinte des paires crâniennes ou cérébrale. L'imagerie clinique est basée essentiellement sur la TDM et l'IRM. Le scanner montre une tumeur lobulée de contours irréguliers, destructrice et de densité inférieure à celle de l'os avec parfois des calcifications. La TDM apporte des précisions sur les destructions osseuses notamment la lame criblée de l'ethmoïde, les parois de l'orbite, le palais osseux et la fosse infra temporale. L'IRM précise l'extension aux tissus mous essentiellement sensoriels et vitaux, elle fait la différence entre les tissus granulomateux et les récidives lors de la surveillance des chondrosarcomes opérés [7]. L'IRM n'est pas faite chez nos malades, elle aurait précisé, surtout chez le deuxième malade, l'importance de l′extension orbitaire et endocrânienne. La biopsie est redue aisée grâce à l'endoscopie endonasale. Le diagnostic de certitude est histologique et immunohistochimique, la différence entre chondrome et chondrosarcome pose des difficultés aux anatomopathologistes [4]. Les chondrosarcomes sont classés en trois grades en fonction de la densité tissulaire, la différenciation nucléaire et la taille du noyau [8]. Les types histologiques sont la variante myxoïde est plus d'origine tissulaire mou que osseux, la variante mésenchymateuse est la plus agressive et survient dans les deux tiers des cas avant 30 ans et un grade avancé [9].

Le traitement est essentiellement chirurgical associé a une radiothérapie malgré la différence des avis sur la radiosensibilité des chondrosarcomes. La chimiothérapie a un rôle limité dans ce traitement, indiquée dans les cas de haut grade de malignité, le chondrosarcome mésenchymateux et dans les récidives ou métastases [10]. La survie à 5 ans est de 44 à 87% [5, 11], les facteurs pronostic sont, l’âge, le grade, le site primitif de la tumeur et la variante myxoide ou mésenchymateuse.

## Conclusion

Le chondrosarcome des sinus de la face est une tumeur rare et agressive, son évolution est lente. L'imagerie clinique est très importante dans toutes les étapes diagnostic, thérapeutique et de surveillance. L'excision chirurgicale est un facteur très important dans la réussite du traitement et la baisse de la fréquence des récurrences.
